# Digital vs face‐to‐face information provision in patient counselling for prenatal screening: A noninferiority randomized controlled trial

**DOI:** 10.1002/pd.5463

**Published:** 2019-05-10

**Authors:** Robert Adrianus de Leeuw, Sabine Fiona Bianca van der Horst, Anneloes Maaike de Soet, Jeroen Patrick van Hensbergen, Petra Cornelia Afra Maria Bakker, Michiel Westerman, Christianne Johanna Maria de Groot, Fedde Scheele

**Affiliations:** ^1^ Athena Institute for Trans‐Disciplinary Research VU University Amsterdam Amsterdam The Netherlands; ^2^ Amsterdam UMC Vrije Universiteit Amsterdam Amsterdam The Netherlands; ^3^ Department of Internal Medicine Franciscus Gasthuis & Vlietland Hospital Rotterdam The Netherlands

## Abstract

**Objective:**

To evaluate face‐to‐face information provision in patient counselling for prenatal screening compared with two forms of digital information provision, namely, noninteractive instructional video or interactive video.

**Method:**

We performed a prospective, noninferiority, cluster‐randomized controlled trial comparing face‐to‐face (usual care) with two forms of digital information provision (intervention) in counselling for prenatal screening. This study was performed in the Amsterdam UMC, the Netherlands, in 2017, and included women in the first trimester of pregnancy. Main outcomes were knowledge gained by the patient and counselling duration. We performed a noninferiority analysis.

**Results:**

One hundred forty‐one women were included, randomized, and analysed. The baseline characteristics were comparable. The intervention group was noninferior compared with the control group regarding the level of satisfaction. The knowledge grade difference was higher after using intervention, and the duration was significantly longer in the face‐to‐face group at 23 minutes versus 16 minutes. The addition of interaction with the video made no difference in any of the outcomes.

**Conclusion:**

Adding an instructional video to patient counselling is of added value to improve patient's knowledge and shorten time consumption of the counsellor, therefore possibly saving costs. But this form of counselling maintains the same level of satisfaction.

## Abstract 2

What is already known about this topic?

- Counselling for prenatal screening is a complex process containing education, information, and evaluation in order to make a well‐considered decision.
- Counselling for prenatal screening has an increase in interdoctor variation and unpredictable time consumption.

What does this study add?

- Digital information provision added to face‐to‐face counselling shortens the counsellors' time significantly without decreasing satisfaction and even improving knowledge.
- Shortening the counsellors' time consumption can be a very cost‐effective way of saving time or increasing atient care.
- Adding interactivity to patient information provision does not improve knowledge or satisfaction.

**What's already known about this topic?**

- Counselling for prenatal screening is a complex process containing education, information, and evaluation in order to make a well‐considered decision.
- Counselling for prenatal screening has an increase in interdoctor variation and unpredictable time consumption.

**What does this study add?**

- Digital information provision added to face‐to‐face counselling shortens the counsellors' time significantly without decreasing satisfaction and even improving knowledge.
- Shortening the counsellors' time consumption can be a very cost‐effective way of saving time or increasing patient care.
- Adding interactivity to patient information provision does not improve knowledge or satisfaction.

## 1 INTRODUCTION

Prenatal screening was developed in the 1970s, as the result of medical innovations, such as invasive prenatal diagnostic tests and obstetrical ultrasound. Since then, prenatal screening has been a routinely offered medical test.^1^ This has led to new challenges, among which is the counselling needed for prenatal screening. Comprehensive prenatal counselling is complicated; it should be personalized and provide sufficient information to promote autonomous decision‐making.^2^ Earlier research has shown not only the importance of counselling but also an increase in intercounsellor variation and unpredictable time consumption, depending on the complexity of the subject.^3^, ^4^

A counselling consultation can be divided into two parts: information provision and the counselling itself. Since the counselling is complex and can have different requirements in different countries, such as face‐to‐face contact,^5^ provision of information is where new innovations may be of help. In a digital age, patients increasingly need digital information provision and even digital counselling.^6^ Previous studies show that websites provide added value beyond face‐to‐face information^7^ and recommend online resources.^8^ Yet they also show that it is difficult and confusing to find information online.^9^ A review from Marokasis et al in 2016 shows that parents desire written, visual, and Web‐based information as soon as possible after prenatal diagnosis.^10^

There is yet another evolving area of innovation, which is the use of interactive electronic media to facilitate teaching and learning.^11^ Better educated patients tend to be healthier, and e‐learning contributes to increased knowledge, improves satisfaction with the consent process and consultation, and is shown to be effective in improving both physical and mental health outcomes.^12^, ^13^, ^14^, ^15^, ^16^ It is unknown whether digital information provision (instructional video with or without interactivity) can be as satisfying as information provided face to face in prenatal counselling during screening for prenatal testing. There is evidence that instructional videos do not have to include interactive elements to be effective.^17^ However, evidence in the field of prenatal screening counselling is lacking.

In this study, we analysed whether an instructional video is noninferior to face‐to‐face information provision with regard to patient satisfaction. We also evaluated if video can be beneficial in regard to learning effect and duration of counselling. Moreover, we aimed to evaluate whether the addition of interactive elements to the instructional video is of added value.

## 2 METHOD

### 2.1 Trial design and participants

We performed a prospective, noninferiority, cluster‐randomized controlled trial comparing face‐to‐face (control group) with digital (intervention group) information provision before counselling for prenatal screening. All eligible patients were invited in the outpatient clinic of the Amsterdam University Medical Centers, VUmc University Amsterdam, the Netherlands. Each consultation consisted of two parts: information provision and followed by face‐to‐face, personal counselling. The control group received usual care, meaning a single consultation of face‐to‐face information provision and counselling. The intervention group was randomized between information provision by means of an instructional or interactive video before they continued to face‐to‐face, personal counselling. Both groups had the same face‐to‐face component. The counsellors (n = 5) were experienced and trained according to the national guidelines (both midwives and doctors follow the same training and perform at least 50 counselling sessions per year), to inform the participants of the same content as that given in the video.

**Figure 1:**
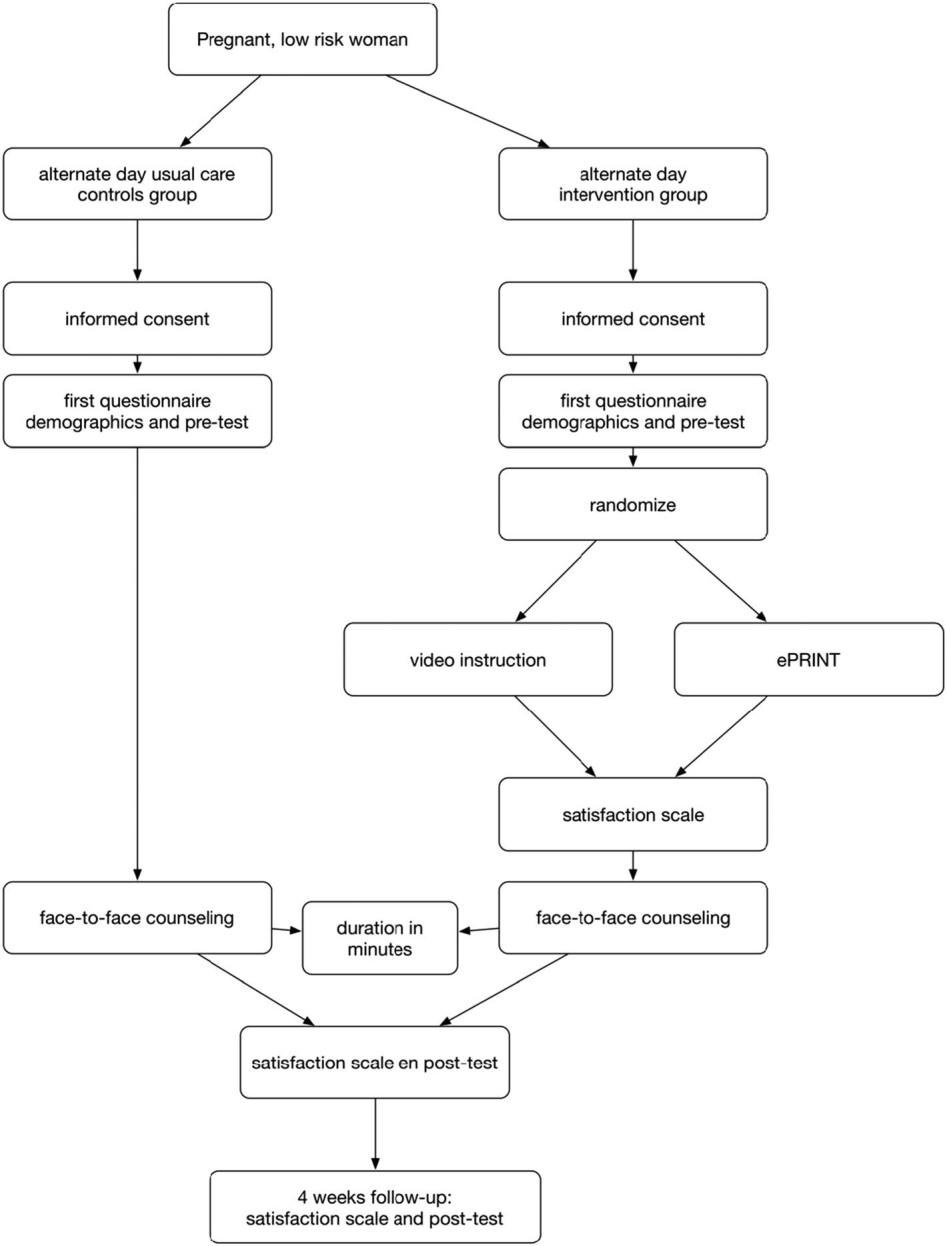
Inclusion flow chart

In the trial, we included healthy women in the first trimester of their pregnancy, aged above 18 years, who had requested counselling for prenatal screening. The participants referred for an indication of increased risk of a chromosome abnormality in pregnancy were excluded. After filling out the informed consent form, the participants were given a short demographics questionnaire; they also completed a knowledge pretest, satisfaction questionnaire afterwards, and knowledge posttest directly after the counselling. The counsellors were blinded for the intervention randomization (instructional or interactive video) outcome and received a satisfaction questionnaire after the counselling. Counsellors timed the duration of their total consultation. The participant watched or interacted with the video in a private waiting room immediately before the counselling. Women were asked to participate when they were 18 years or older, spoke the Dutch language, and came for routine prenatal screening counselling. They were excluded when they had an increased risk on chromosomal abnormalities. Thirty‐four women did not meet the inclusion criteria, mainly because of insufficient Dutch language skills. One hundred participants were lost in follow‐up at the 6‐week questionnaire, after which we decided not to use the related data for further interpretation. See Figure 1 for the inclusion flow chart.

### 2.2 Information provision

Information provision and the counselling should usually be followed by a well‐informed decision on the part of the participant. All groups received both the same information and counselling in one consultation. The standardized information provision consisted of general information about the basic understanding of prenatal screening, the screening options, and the possible consequences of a negative or positive test result. The aim of the information provision was to educate the participant to enable her to make a proper, well‐informed decision during the counselling. This study addresses the knowledge participants gain and the satisfaction of the consultation, which are part of being well informed. After the counselling, we wished the participants to have insight into the following subjects: prevalence of trisomies in the general Dutch population, which chromosomal anomalies are tested for and how, which screening methods are offered, the difference between screening and diagnosing, the difference between invasive and noninvasive testing, and finally, the limitations of ultrasound screening. The video was based on a previously used group consultation presentation.

Both the information provision and the counselling are needed to decide whether to undergo prenatal screening. Therefore, we carried out a pretest before the information provision and a posttest after the counselling. The key differences between the interactive and passive video were the mandatory questions that pause the interactive video. We added a progress bar, four pauses with written information, 10 multiple‐choice questions, and five stop/rewind popups elements to the video. Please find the instructional and interactive video here: http://eprint.elephantelearning.com/video‐page/

### 2.3 Randomization

Counselling took place 2 days per week. Because of logistics in the outpatient clinic, it was not possible to randomize all subjects every day, and we had random days of usual care and days of video information provision. On the video provision days, we computer randomized immediately before the counselling between an instructional (noninteractive) and an interactive video. The allocation was 1:1 for the control group and the intervention group and, again, 1:1 for the instructional video and the interactive video group. Participants were given an appointment at the desk for either of those days randomly (the administrative personnel were blinded). All counsellors were blinded for the randomization outcome.

### 2.4 Outcomes

Our primary outcome was the level of participant satisfaction after the entire consultation. Satisfaction was preferred over knowledge as primary outcome, because in case of counselling, it is more important to make a well‐informed, satisfying decision than to gain knowledge of the subject.^18^ Satisfaction is determined by a previously validated, adjusted and translated Genetic Counselling Satisfaction scale..^18^ The secondary outcomes were the duration of the face‐to‐face counselling that followed, the blinded counsellors' satisfaction with the consultation on a scale of 1 to 5, and the participant knowledge score before and after the counselling. Knowledge was evaluated by means of a seven‐question test based on the content of the information provided.

### 2.5 Statistical analysis

Data collection was conducted using research survey^19^ as a data management system, and data analysis and reporting were carried out according to the CONSORT guideline^20^ with IBM SPSS as statistical analysis software. A descriptive table for the baseline characteristics is reported and primary, and secondary outcomes were analysed on a noninferiority basis. Statistical analysis was conducted using SPSS for Windows version 10.0 on PC computer. None of the results were normally distributed; thus, no transformations were applied. The main outcomes of the survey were compared using the Aspin‐Welch test. Difference within groups was analysed using the Wilcoxon signed‐rank test. The analysis was conducted in two phases. Phase one was a comparison between the main groups, and phase two compared the intervention subgroups. Fisher test on binary outcomes could not be conducted since one of the groups had a 100% score. We calculated that a sample size of 160 woman (40 participants per video group and 80 in the control group) would be needed, with a risk of type I error of 5% and a power of 80%, to show statistically significant difference in satisfaction. We hypothesized that the group difference could be around 18%, based on earlier participant education studies (on premature birth).^12^ We added 25% lost in follow‐up; therefore, we needed 200 participants in total.

## 3 RESULTS

**Figure 2:**
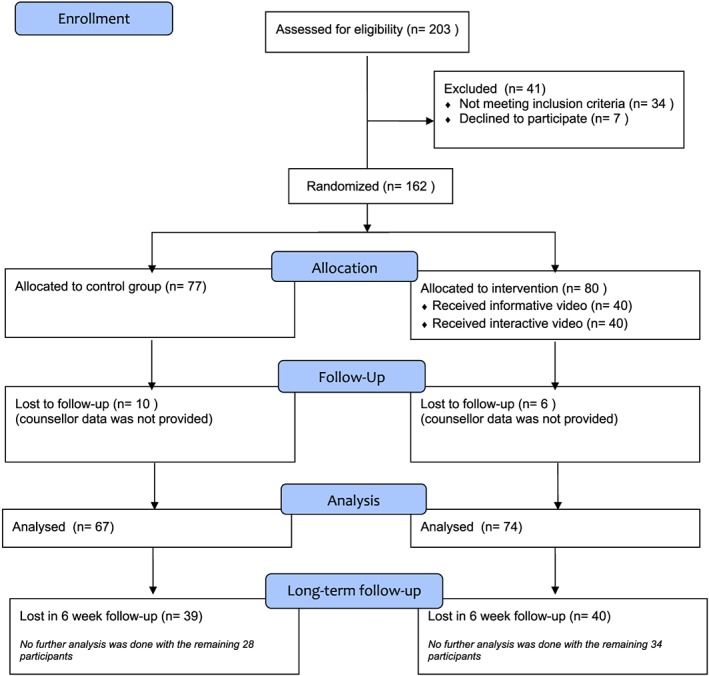
CONSORT flow chart [Colour figure can be viewed at wileyonlinelibrary.com]

Between August 2017 and December 2017, two hundred three women were approached and asked to participate. We stopped including participants when the sample size was reached. One hundred and sixty‐two women consented, and five were lost in follow‐up for the postcounselling questionnaire. These five participants (all in the control group) did not fill out their postcounselling questionnaire. We lost 10 participants in the control group and six in the intervention group because the counsellor did not fill out their questionnaire about these participants. We were able to analyse 67 women in the control group, 36 in the informative video group, and 38 in the interactive video group for the primary outcomes (Figure 2, CONSORT flow chart). We formulated higher education as a completed bachelor or master's degree. We asked the participants to score their presumed knowledge on a scale of 1 to 10 (self‐assed knowledge score). Overall, almost one‐third of the participants were primiparous, less than half had previous experience with prenatal screening, almost 80% were higher educated, and more than half had a positive attitude towards prenatal screening.

The baseline characteristics were comparable between the groups, with the exception of the positive attitude towards screening; more women were positive in the intervention group towards screening (Table 1). There were no differences between the instructional video and interactive video groups (Table 2). Although there were more participants in the intervention group with previous experience with prenatal screening.

**Table 1:** Demographics usual care group and intervention

	Control Group	Intervention Group
	n = 77	n = 80
Maternal age (average) at inclusion	33.6 ± 4.5	35.1 ± 4.1
Married or cohabiting (% yes)	76 (98.7%)	75 (93.8%)
Education (% higher educated)	58 (75.4%)	65 (81.2%)
Multi para (% yes)	55 (71.4%)	56 (70%)
Experience with prenatal screening (% yes)	25 (45.5%)	34 (60.7%)
Attitude towards screening (%positive)	34 (44.2%)	57 (71.3%)
Companion at consultation (% yes)	56 (72.7%)	51 (63.7%)
Education of companion (% higher educated)	36 (64.3%)	33 (64.7%)
Self‐assessed knowledge score (average on 1‐10)	5.62 ± 2.463	6.87 ± 1.580
Religious (% yes)	30 (39%)	24 (30%)

**Table 2:** Demographics of the intervention group

	Intervention Group	
	Instructional Video	Interactive Video
	n = 40	n = 40
Maternal age (average)	35.6 ± 4.7	34.7 ± 3.4
Married or cohabiting (% yes)	38 (95%)	37 (92.5%)
Education (% higher educated)	37 (92.5%)	28 (70%)
Multi para (% yes)	26 (65%)	30 (75%)
Experience with prenatal screening (% yes)	17 (65.4%)	17 (56.7%)
Attitude towards screening (%positive)	31 (77.5%)	26 (65%)
Companion at consultation (% yes)	25 (62.5%)	26 (65%)
Education of companion (% higher educated)	21 (84%)	12 (46.1%)
Self‐assessed knowledge score (average on 1‐10)	7.13 ± 1.418	6.63 ± 1.705
Religious (% yes)	10 (25%)	14 (35%)

### 3.1 Primary and secondary outcomes

There was no difference between the control group and the intervention group in the satisfaction scale nor among the subgroups of the Counselling Satisfaction scale, 3.91 (CI, 3.38‐4.42) and 3.93 (CI, 3.53‐4.33), *P* = .88, respectively (Table 3). In both groups, over 95% of the participants were satisfactorily informed (95.6% control group and 100% intervention group). We asked participants if they already made a choice considering the prenatal screening before and after the counselling, and 5.9% changed their opinion in the control group, while 7.2% changed their opinion in the intervention group (of those who changed their mind, 94% changed from wanting prenatal screening to no longer wanting screening).

**Table 3:** Primary outcomes

Outcome	Control	Intervention	P Value
Participant satisfaction	n = 68	n = 69	
Genetic Counselling Satisfaction scale	3.9 ± 0.5	3.9 ± 0.4	.88
Satisfactorily informed (% yes)	95.6	100	
Knowledge grade before counselling	5.7	6.1	.11
Knowledge grade difference pre/post test	+0.91	+2.07	.00
Counsellor outcomes	n = 58	n = 63	
Duration of counselling (average minutes)	23.0 ± 6.6	16.3 ± 7.4	.00
Counsellor satisfaction (scale 1‐10, average)	8.0 ± 1.2	7.7 ± 0.9	.172
Counsellor satisfaction (Likert 1‐5, average)	4.2 ± 0.7	4.1 ± 0.7	.393
Well‐considered decision (scale 1‐5, average)	4.4 ± 0.7	4.3 ± .08	.282

The duration of the counselling was 7 minutes shorter in the intervention (*P* = .00). After counselling, the knowledge grade increased in both groups. However, the difference between precounselling and postcounselling knowledge grade was significantly greater in the intervention group. The knowledge score before the counselling was not significantly different between the control and intervention groups. Counsellor satisfaction after the counselling was not significantly different.

### 3.2 Subgroup analysis instructional video versus interactive video

Comparing the instructional video group with the interactive video group reveals no significant difference for any of the outcomes (Table 4). Satisfaction was comparable, as was counselling duration, knowledge grade before and after the counselling, and counsellor's satisfaction.

**Table 4:** Subanalysis

Outcome	Passive Video	Interactive Video	P Value
Participant satisfaction	n = 33	n = 36	
Genetic Counselling Satisfaction scale	4.0 ± 0.5	3.9 ± 0.4	.406
Satisfactorily informed (% yes)	100	100	nnvt
Knowledge grade before counselling	6.07	6.14	.85
Knowledge grade difference pre/post test	2.1 ± 1.7	2.1 ± 1.6	.987
Follow‐up 4‐6 weeks	n=15	n=19	
Satisfaction (scale 1‐5, average)	3.5 ± 1.2	3.5 ± 0.7	.984
Well‐considered decision (scale 1‐5, average)	3.9 ± 1.3	4.0 ± 0.6	.716
Counsellor outcomes	n=29	n=34	
Duration of counselling (average minutes)	17.1 ± 7.5	5.6 ± 7.3	.432
Counsellor satisfaction (Likert 1‐5, average)	4.1 ± 0.7	4.1 ± 0.7	.81
Well‐considered decision (scale 1‐5, average)	4.4 ± 0.8	4.2 ± 0.8	.226

## 4 DISCUSSION

### 4.1 Main findings

Using digital information provision as part of the counselling for prenatal screening proves to be just as satisfying as face‐to‐face information provision. The counsellors spent an average of 7 minutes less per consultation and the participants using digital information provision performed better on the knowledge test. Adding interactivity to the video had similar results to the noninteractive video.

### 4.2 Strengths and limitations

This study shows that the use of video in patient information provision before prenatal screening counselling is acceptable for patients and has a potential benefit for the counsellor. When evaluating an instructional instrument, it is crucial to have a control group and, if possible, to evaluate an educational variation as well. This study did both. Furthermore, the study reached the necessary sample size to evaluate our primary outcome: participant satisfaction. Finally, randomization was ensured, given the comparable baseline characteristics. There were also limitations. First of all, we were not able to randomize by participant; however, we randomized days between the control group and the intervention. This could cause a selection bias, although we blinded the administration and participants, and it did not affect the baseline characteristics. Although the baseline characteristics did show that the study population is very homogenous and overall very well educated. This will influence the generalizability of the results. Secondly, the primary outcome, satisfaction is, because of its subjective nature, a difficult outcome to measure. It could very well be that participants filled out a satisfaction scale more positively when they had invested more time in watching a video. Our secondary outcome knowledge was not corrected for a possible test retest bias, which could have caused an increase in knowledge but should be expected to be equal on both groups. A long‐term follow‐up and a qualitative evaluation can be performed to have better insight in whether the participant's decision was in line with her values (the value‐based decision‐making of the participant). Thirdly, we were not able to have a proper 6‐week follow‐up and even had a loss in the counsellor questionnaire in follow‐up. We believe this is due to the fact that participants, and even counsellors, did not have enough commitment for this study to invest more time for the follow‐up. Finally, we did not properly pilot‐test to evaluate the video and the interactive version before using them. Therefore, improving the design of the intervention (by using the feedback from a pilot) could have improved the outcomes of the video and, especially, of the interactive video. This study can be interpreted as using a pilot instrument, of which we can work on further improvement.

### 4.3 Interpretation

Patient counselling is becoming increasingly important in shared decision making, and this study shows that digital media can make it more time effective and cost‐effective. The benefit of using an instructional video is that each participant will get the same information. Face‐to‐face information provision never guarantees this.^13^ This could be the reason that the intervention group performed better on the knowledge test. Of course, the counselling of patients who had already been informed by video was shorter than that given to the patients in the control group. This is the most logical consequence of adding the intervention to the counselling. It is good to realize that prenatal counselling is complex, and the needs are manifold,^4^ but digital information provision does improve knowledge, shortens the counselling, and remains the same level of satisfaction. Adding an instructional video to the consultation made it more time consuming for the participants, with equal satisfaction and more knowledge gain. Although the counsellor was equally satisfied, it seems that their satisfaction is not influenced by the duration of the counselling. These 7 minutes per counselling can save a lot of money. In the Netherlands, we have 170 000 woman per year that get pregnant. If we would counsel all these woman, 7 minutes shorter, at a 150 euro per hour fee, we can save 2.9 million euro per year in the Netherlands. A proper cost‐effectiveness study should be performed to support this claim. The knowledge gain is in line with other studies that show the added value of computer‐assisted instruction in improving patients' knowledge and satisfaction.^21^ Yee et al show that an interactive computer aid can convey the relevant information about genetic screening and diagnostic concepts,^22^ although they did not evaluate this within the context of counselling and their focus was on knowledge only.

The fact that adding interactivity to the video did not improve the experience is also in line with other studies showing inconsistent results from adding interactivity.^23^ A recent study by Logan et al shows that an instructional video benefits most from segmenting and least by adding practice and inserting pauses.^17^ The difference between our instructional and interactive video mainly contains segmenting, practices, and pauses. The lack of effect is therefore in line with Logan et al. We believe that future studies will have additional value when evaluating more of these variations. Another reason could be the already fulfilled engagement. A theory behind the added value of interactivity is the added engagement of the learner towards the topic.^24^ Perhaps, the emotional investment in the topic and the “just‐in‐time” manner of the video already engaged the participant, after which the interactivity is of little added value. Counselling will always need a face‐to‐face element, because of legislation and ethical considerations, but optimizing the patient's prior knowledge, understanding, and attitude can improve counselling. Finding the right format will be the challenge. Whether this will be interactive information aids, virtual reality question‐and‐answer environments or any other variant of digital information provision should be further evaluated. What should also be addressed are the actual implementation challenges of an intervention like this. Can people watch the video at home? Will they all actually watch it? How can the counsellor prevent an unexpected delay when people have not watched it? These are questions that can be evaluated by a follow‐up implementation study.

## 5 CONCLUSION

Our study shows that adding an instructional video to patient counselling is of added value to the time consumption of the counsellor, can save costs, and improves patient's knowledge but maintains the satisfaction of both patient and counsellor. Adding interactivity to the instructional video did not change these effects. There is still much to learn about patient counselling and education. Other methods should be evaluated, other educational strategies can be used, and further evaluation studies should always include an instructional design evaluation as well.

## CONFLICT OF INTEREST

None of the authors have any conflict of interest.

## AUTHOR CONTRIBUTION

R.D.L., P.C.B. and C.J.G. conceptualized the trial. Together with S.F.H., A.M.S. and F.S., they drafted the protocol, which was revised by all authors. R.D.L., S.F.H., and A.M.S. included all participants. R.D.L., S.F.H. and J.H. performed the statistical analysis. All authors read and approved the final manuscript.

## DETAILS OF ETHICAL APPROVAL

The study protocol was approved by the medical ethical committee of the VU University, Amsterdam, the Netherlands, with file ID 398 on August 2th 2017.

## DATA AVAILABILITY STATEMENT

Data are available on request from the authors.
